# Ancient Adaptive Evolution of the Primate Antiviral DNA-Editing Enzyme APOBEC3G

**DOI:** 10.1371/journal.pbio.0020275

**Published:** 2004-07-20

**Authors:** Sara L Sawyer, Michael Emerman, Harmit S Malik

**Affiliations:** **1**Division of Basic Sciences, Fred Hutchinson Cancer Research CenterSeattle, Washington, United States of America; **2**Human Biology, Fred Hutchinson Cancer Research CenterSeattle, WashingtonUnited States of America

## Abstract

Host genomes have adopted several strategies to curb the proliferation of transposable elements and viruses. A recently discovered novel primate defense against retroviral infection involves a single-stranded DNA-editing enzyme, APOBEC3G, that causes hypermutation of HIV. The HIV-encoded virion infectivity factor (Vif) protein targets APOBEC3G for destruction, setting up a genetic conflict between the *APOBEC3G* and *Vif* genes. This kind of conflict leads to rapid fixation of mutations that alter amino acids at the protein–protein interface, referred to as positive selection. We show that the *APOBEC3G* gene has been subject to strong positive selection throughout the history of primate evolution. Unexpectedly, this selection appears more ancient than, and is likely only partially caused by, modern lentiviruses. Furthermore, five additional *APOBEC* genes in the human genome appear to be engaged in similar genetic conflicts, displaying some of the highest signals for positive selection in the human genome. Despite being only recently discovered, editing of RNA and DNA may thus represent an ancient form of host defense in primate genomes.

## Abstract 2

APOBEC3G, a gene that edits retroviral DNA like HIV, is under positive selection that predates the origin of HIV, implying that RNA/DNA editing represents an ancient form of intragenomic host defense

## Introduction

Mobile genetic elements have been in conflict with host genomes for over a billion years. Our own genomes reveal the remarkable effects of retrotransposition, as about 45% of our genomic DNA results directly from this process (Lander et al. 2001). This perennial state of conflict has led eukaryotes to adopt several strategies to curb the proliferation of transposable elements and viruses. These include transcriptional silencing through DNA and histone methylation (Tamaru and Selker 2001; Selker et al. 2003) or RNA interference (Ketting et al. 1999; Tabara et al. 1999; Aufsatz et al. 2002), and even directed mutagenesis of mobile elements (Selker et al. 2003). Despite facing this gauntlet of defense strategies, transposable elements have thrived in eukaryotic genomes (with Neurospora crassa being a notable exception [Selker et al. 2003]) by evolving suitable countermeasures. Our current understanding of the intracellular interplay between host defenses and the assault of transposable elements suffers from a paucity of cases where both counterstrategies have been clearly identified. This is in contrast to extracellular cases, where interactions between viral proteins and either host immune surveillance or host receptors have been well established. Understanding the nature and evolutionary time-frame of intracellular conflict is key to understanding the current state of eukaryotic genomes. Recent studies of host inhibition of HIV have uncovered mutations introduced by DNA editing as a novel means by which host genomes battle viruses intracellularly. Furthermore, the means by which viruses combat this defense strategy are also identified, thus providing an unprecedented opportunity to study the evolution of intracellular genetic conflict.

Different human cell lines vary in their susceptibility to HIV infection. The gene responsible for this differential susceptibility was identified as *apolipoprotein B–editing catalytic polypeptide 3G (APOBEC3G)* (Sheehy et al. 2002), whose product targets HIV and simian immunodeficiency virus (SIV) for editing as their genomes undergo reverse transcription in the cytoplasm of host cells. APOBEC3G is a cytidine deaminase that edits cytosines to uracils in the minus strand DNA copied from the viral RNA genome, resulting in promiscuous guanine-to-adenine (G-to-A) hypermutation of the plus (protein-coding) strand of the viral DNA (Harris et al. 2003; Mangeat et al. 2003; Zhang et al. 2003). APOBEC3G is expressed in testes, ovary, spleen, peripheral blood leukocytes, and T-lymphocytes (Jarmuz et al. 2002; Sheehy et al. 2002) and is packaged in nascent virions and delivered into new host cells along with the viral genome (Harris et al. 2003). How this editing reduces the evolutionary fitness of the virus is not well established. The mutations introduced by the editing process may either directly reduce viral fitness, or target the uracil-containing viral DNA for destruction (Gu and Sundquist 2003). Before the discovery of APOBEC3G, RNA editing was thought to function solely in the diversification of gene-encoded information. The discovery of viral targeting by APOBEC3G represents a new phase in our understanding of nucleic acid editing in primates.

**Figure 1:**
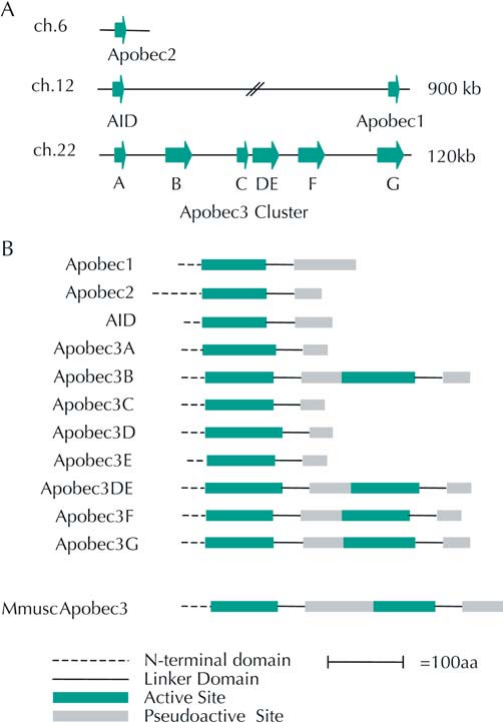
The Primate *APOBEC* Family (A) The human genome contains nine known members of the *APOBEC* family. *AID* and *APOBEC1* are located approximately 900 kb apart on human Chromosome 12. The primate-specific *APOBEC3* cluster of six genes resides on human Chromosome 22, and likely arose through a series of gene duplication events (Jarmuz et al. 2002; Wedekind et al. 2003). The single *APOBEC3*-like gene found in mouse resides on Chromosome 15 (not shown), which is syntenic to human Chromosome 22 (Sheehy et al. 2002). There is EST evidence for both APOBEC3D and APOBEC3DE (see Materials and Methods), and we treat these as three separate transcripts in our analysis because currently there is no evidence for the relevant protein products. (B) All members of the APOBEC family contain an active site that encodes a zinc-dependent cytidine deaminase domain with the HXE, PCXXC signature (Mian et al. 1998), a linker peptide, and a pseudoactive domain (Navaratnam et al. 1998; Jarmuz et al. 2002). The active and pseudoactive domains are related by structure only, and likely originated from a gene duplication event followed by degeneration of the catalytic activity of the pseudoactive domain. Several members of the human *APOBEC3* gene cluster (*APOBEC3B, 3DE, 3F,* and*3G*) have undergone an additional duplication/recombination event and now contain two each of the active and pseudoactive sites (Jarmuz et al. 2002; Wedekind et al. 2003), as does the single *APOBEC3*-like gene found in mouse. DOI:10.1371/journal.pbio.0020275.g001

*APOBEC3G* belongs to a family of nine primate genes that catalyze the deamination of cytosine to uracil in DNA and/or RNA (Figure 1). Two other members of this family are known to have important in vivo editing functions. *APOBEC1* encodes a protein that site-specifically edits the mRNA of *apolipoprotein B (APOB),* leading to a truncated form of the APOB lipid-transport protein (Chan et al. 1997), which is important for determining levels of low-density lipoprotein production. Another member of this family, activation-induced deaminase (AID), is important for all steps following V(D)J recombination in B lymphocytes (Fugmann and Schatz 2002), from generating antibody diversity to class-switching events. Significantly, APOBEC1 and AID act within the nucleus, whereas APOBEC3G is exclusively cytoplasmic, which prevents it from mutating “self” DNA molecules. Whereas rodents have a single *APOBEC3* gene, humans have at least six (Jarmuz et al. 2002), including *APOBEC3G*. The functions of the other members of this expanded *APOBEC3* cluster are unknown, although APOBEC3C has been shown to be catalytically active, exhibiting DNA mutator activity in a bacterial system that is like APOBEC3G (Harris et al. 2002). More recently, APOBEC3F has also been associated with anti-HIV biological activity (Wiegand et al. 2004; Zheng et al. 2004).

Most lentiviruses encode an accessory gene, *virion infectivity factor (Vif),* whose product counteracts the antiviral activity of APOBEC3G. Vif interacts with APOBEC3G and targets it for ubiquitination and proteasome-dependent degradation, thus preventing its incorporation into nascent virions (Madani and Kabat 1998; Simon et al. 1998; Marin et al. 2003; Sheehy et al. 2003; Stopak et al. 2003; Yu et al. 2003). This interaction can be species-specific, as the Vif protein of one lentivirus will counteract APOBEC3G from its host species, but not always the APOBEC3G from a different primate species (Mariani et al. 2003). Thus, *APOBEC3G* and *Vif* are predicted to be under selection to decrease and enhance, respectively, their interaction with one another, each driving rapid change in the other. Genetic conflicts like this one are predicted to result in the rapid fixation of mutations that alter amino acids, specifically those that affect this protein–protein interaction. This scenario is referred to as positive selection and is commonly seen in host–pathogen interactions.

In this report, we directly test this prediction by studying the paleontology of selective pressures that have acted on *APOBEC3G* in the primate lineage, to ask whether *APOBEC3G* has been subject to positive selection, and to date the origins of this genetic conflict. We find that *APOBEC3G* has been under remarkably strong positive selection, and has undergone several episodes of adaptive evolution throughout the history of primates. Unexpectedly, we find that the positive selection acting on *APOBEC3G* predates modern lentiviruses, indicating that a more ancient, and perhaps ongoing, conflict has shaped its evolution. We also report evidence for strong positive selection acting on a majority of the *APOBEC* genes, suggesting that this family of genes may have expanded in primate genomes for genome defense via RNA/DNA editing.

## Results/Discussion

### *APOBEC3G* Has Been Evolving under Positive Selection in Primates

**Figure 2:**
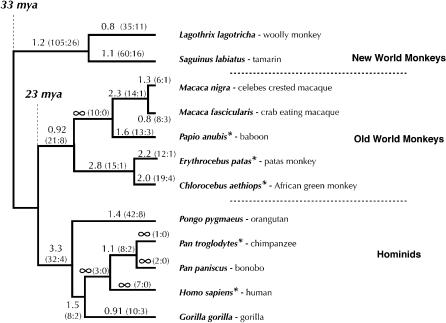
APOBEC3G Has Been Under Positive Selection for at Least 33 Million Years The ω values and actual numbers of non-synonymous and synonymous changes (R:S, included in parentheses) in APOBEC3G are indicated on the accepted primate phylogeny (Purvis 1995) that includes five hominids, five OWMs, and two NWMs. OWMs diverged from hominids about 23 million years ago, whereas NWMs diverged around 33 million years ago (Nei and Glazko 2002). ω values were calculated using the PAML package of programs using the free-ratio model that allows ω to vary along each branch. In some instances, zero synonymous substitutions lead to an apparent ω of infinity. HIV/SIV-infected species are indicated by asterisks. DOI:10.1371/journal.pbio.0020275.g002

To determine what selective pressures have shaped *APOBEC3G* evolution, we sequenced the *APOBEC3G* gene from a panel of primate genomes representing 33 million years of evolution. We sequenced the complete *APOBEC3G* coding sequence (approximately 1,155 bp) from ten primate species, including four hominids (other than human), four Old World monkeys (OWMs), and two New World monkeys (NWMs) (Figure 2). A phylogeny constructed using either complete *APOBEC3G* sequences or individual exons (unpublished data) is congruent to the widely accepted primate phylogeny (Purvis 1995), indicating that all sequences isolated by our PCR strategy are truly orthologous.

The hallmark of positive selection is an excess of non-synonymous substitutions (which alter the amino acid being encoded) relative to synonymous substitutions (which retain the encoded amino acid). Because non-synonymous changes are more likely to be deleterious, they are typically culled out by selection (Hurst 2002) (referred to as purifying or negative selection). Therefore, in protein-coding open reading frames, the number of observed changes per synonymous site (Ks) usually exceeds the number of observed changes per non-synonymous site (Ka). In the case of the *APOBEC3G,* however, we found that a majority of branches of the phylogeny (including internal branches) show evidence of positive selection (defined as Ka/Ks [ω] greater than one; see Figure 2). This implies that the *APOBEC3G* has been subject to positive selection throughout the history of primate evolution. In support of this conclusion, *all* pairwise comparisons of the entire *APOBEC3G* gene between the various primates have ω greater than one (unpublished data). Maximum likelihood analysis using the PAML (phylogenetic analysis by maximum likelihood) suite of programs also finds strong evidence that the full-length *APOBEC3G* gene has been subject to positive selection (*p* < 10^–13^). Numbers in parenthesis in Figure 2 indicate the actual number of non-synonymous and synonymous changes (R:S) that have occurred along each branch.

The average Ks in *APOBEC3G* is not unusually low; it is about 0.09 between hominids and OWMs and 0.26 between hominids and NWMs, compared to 0.08 and 0.15 respectively for comparisons of various intronic and noncoding regions of primate genomes (Li 1997). Thus, we can rule out the possibility that selection has led to deflated Ks values in *APOBEC3G* that lead to artificially high ω ratios. Indeed, these high ω ratios can be explained only by a significantly higher rate of non-synonymous substitutions. Of the primates analyzed, lentiviral infections have been observed only in the African monkeys, chimpanzees, and humans (Peeters and Courgnaud 2002). HIV/SIV-infected species are indicated with asterisks in Figure 2. Estimating the age of lentiviruses is difficult because of their rapid rate of evolution and frequent cross-species transfer, but it has been suggested that primate lentiviruses are no older than 1 million years (Sharp et al. 1999). The presence of modern lentiviruses appears to bear no correlation to either the presence or the strength of positive selection. For instance, the lineage leading to hominids has a ω of 3.3, the highest overall. The positive selection acting on *APOBEC3G* thus appears to predate modern lentiviruses, and interactions with lentiviral Vif proteins are not likely to be a major cause of this unusually strong signal of positive selection. In support of this conclusion, HIV has not been in the human population long enough to account for the positive selection of *APOBEC3G* specific to the human lineage (a 7:0 R:S ratio) arguing that, although the positive selection of *Vif* may be explained in large part by that of *APOBEC3G,* the reverse is certainly not the case.

### Positive Selection in *APOBEC3G* Is Not Localized to One Domain

We wanted to identify the specific domains in *APOBEC3G* that were subject to positive selection, because this might suggest the driving evolutionary force. For instance, the positive selection in the major histocompatibility complex proteins is confined to only small segments of the protein that constitute the antigen-recognition site (Hughes and Nei 1988; Yang and Swanson 2002), because only these sites participate in protein–protein interactions subject to genetic conflict. All members of the APOBEC family contain a similar domain organization (see Figure 1B) that consists of an active site that encodes a zinc-dependent cytidine deaminase domain with the HXE, PCXXC (H, histidine; X, any amino acid; E, glutamic acid; P, proline; C, cysteine) signature (Mian et al. 1998), a linker peptide, and a pseudoactive domain (Navaratnam et al. 1998; Jarmuz et al. 2002). The active and pseudoactive domains are believed to have originated from a gene duplication event followed by degeneration of the catalytic activity of the pseudoactive domain. *APOBEC3G* and some other *APOBEC* genes have also undergone a second gene duplication/fusion event (Jarmuz et al. 2002; Wedekind et al. 2003).

**Figure 3:**
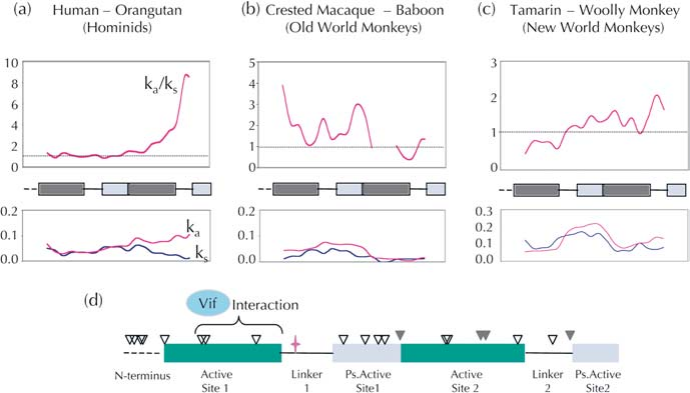
Episodic Positive Selection on Different Regions of the *APOBEC3G* Gene (A–C) Sliding window (300-bp window; 50-bp slide) analysis of Ka and Ks was performed on three representative pairs of primate APOBEC3G sequences, between two hominids (human–orangutan) (A), between two OWMs (crested macaque–baboon) (B), and between two NWMs (tamarin–woolly monkey) (C). Ka/Ks, Ka, and Ks are plotted against the length of the gene (with a schematic of protein domains along the x-axis) to illustrate that different domains of APOBEC3G have undergone positive selection, depending on which lineage is examined. The value for ω, indicated by Ka/Ks, is not shown for part of the crested macaque–baboon comparison (B), because Ks is zero in this region (see plot below). (D) A schematic of the domains of human APOBEC3G illustrates the N-terminal domain (aa 1–29), the two active sites (aa 30–120 and 215–311), and the pseudoactive sites (aa 162–214 and 348–384). Also illustrated is the Vif-interaction domain of APOBEC3G (aa 54–124) (Conticello et al. 2003) as well as the single amino acid residue responsible for species-specific sensitivity to Vif (aspartic acid 128; cross shape in linker 1) (Bogerd et al. 2004; Schrofelbauer et al. 2004). PAML (Yang 1997) was used to identify individual residues (codons) that have significant posterior probabilities of ω greater than 1.0 (see Materials and Methods). Those codons with posterior probabilities greater than 0.95 and greater than 0.99 are indicated by open and closed inverted triangles, respectively (listed in Figures S2 and S3). This represents only a subset of the residues that are likely to be under positive selection, highlighting those residues that have repeatedly undergone non-synonymous substitutions. For instance, residue 128 is not highlighted, as it has a posterior probability of only 0.55 because it has undergone only one fixed non-synonymous change (along the OWM lineage). Domains have been defined by protein sequence alignment to APOBEC1 (Jarmuz et al. 2002). The first pseudoactive domain is likely to include in its C-terminus a second duplication of the N-terminal domain, although this boundary cannot be resolved because of sequence divergence. DOI:10.1371/journal.pbio.0020275.g003

Representative examples of pairwise (sliding window) comparisons of Ka/Ks ratios between two hominids, two OWMs, and two NWMs suggest that the same domain of *APOBEC3G* has not been subject to positive selection throughout primate evolution (Figure 3A–3C). In both the hominid and NWM comparisons, the second half of the gene shows evidence of positive selection (Figure 3A and 3C), but in an OWM comparison, it is the first half that is under positive selection (Figure 3B). When the *APOBEC3G* gene is divided into structural domains, we find that all domains, including the active site domains, have undergone multiple distinct episodes of positive selection (Figure S1). This highly unusual pattern suggests that the genetic conflicts that have shaped APOBEC3G evolution have involved episodic protein–protein interactions with different parts of the *entire* APOBEC3G protein.

We also employed a maximum-likelihood approach (see Materials and Methods), using the PAML suite of programs (Yang 1997) to identify the specific residues that have been repeatedly subject to positive selection in primates. These analyses (in the best fit model) identify 30% of the codons as having evolved under stringent purifying selection (ω of approximately zero). These include the catalytically important residues that are invariant throughout all APOBECs. The same analysis also identifies approximately 30% of the codons as having evolved under positive selection with an average ω of nearly 3.5 (residues that are evolving without selective constraint would be expected to have an average ω of one). Even among adaptively evolving proteins, this is an unusually high proportion of sites, once again implicating a large number of residues in APOBEC3G as having participated in some kind of genetic conflict. Of these, several residues are identified as being under positive selection with high confidence (posterior probability greater than 0.95, inverted triangles in Figure 3D). In simulations using datasets with comparable levels of sequence divergence and strength of positive selection to our *APOBEC3G* dataset (tree length = 1.59), PAML analyses were found to be highly accurate in identifying residues subject to positive selection (Anisimova et al. 2002).

The schematic in Figure 3D highlights the region where Vif is believed to interact with human APOBEC3G (Conticello et al. 2003). It also highlights the single amino acid residue (cross symbol in linker 1) that is responsible for the species-specific interactions seen between Vif and APOBEC3G in African green monkeys (SIV) and humans (HIV) (Bogerd et al. 2004; Schrofelbauer et al. 2004). There is a noticeable lack of correlation between the sites on APOBEC3G that are important for Vif interaction and those sites that are identified by PAML with high confidence, supporting our earlier conclusion that Vif interactions have played only a small role in dictating the positive selection of *APOBEC3G*.

### Other *APOBEC* Genes May Participate in Host Defense

The discovery that *APOBEC3G* is involved in host defense was predicated on the tissue-specific inhibition of HIV. Other studies have investigated a possible inhibitory role of other *APOBEC* genes but found that only *APOBEC3G* and *APOBEC3F* exert an antiviral defense against HIV (Mariani et al. 2003; Wiegand et al. 2004; Zheng et al. 2004). An unbiased look at selective pressures among other *APOBEC* genes could reveal clues to their function. We calculated whole-gene Ka/Ks ratios for other members of the human *APOBEC* family, using orthologs from the chimpanzee genome project (Table 1, second column). This analysis reveals strong evidence of purifying selection acting on *AID* and *APOBEC3A* but positive selection acting on *APOBEC3B* and *APOBEC3DE* (as well as *APOBEC3D* and *APOBEC3E* alone) in addition to *APOBEC3G*. There is no expression evidence for *APOBEC3E,* and it is unclear whether it occurs as a stand-alone gene, but its ω ratio of 5.6 is among the highest seen for *any* human–chimp comparison and argues strongly that it is a functional gene and an active participant in some form of genetic conflict. Whole-gene analyses are notoriously poor at identifying specific domains of positive selection, especially when the rest of the gene is subject to purifying selection. We therefore performed a sliding window Ka/Ks test (Endo et al. 1996), which also reveals positive selection acting on *APOBEC3F* (amino acids [aa] 117–250).

**Table 1:**
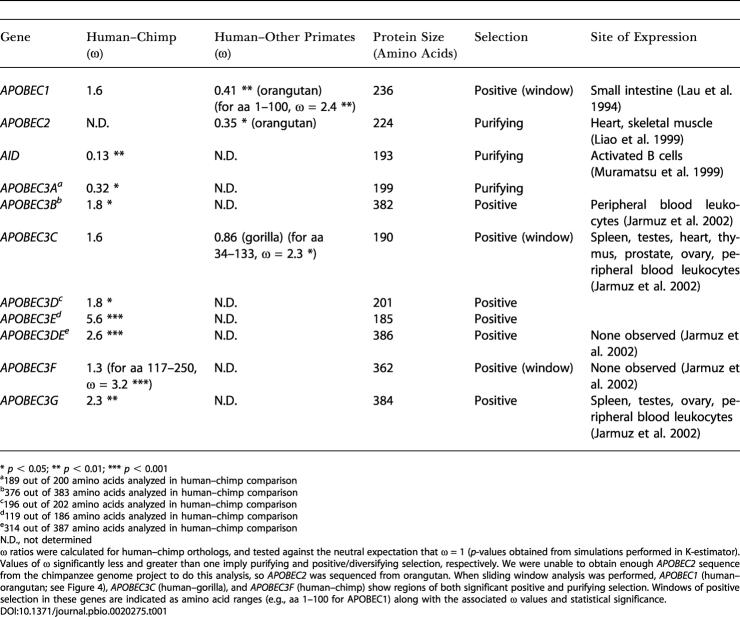
Positive Selection throughout the *APOBEC3* Gene Cluster

* *p* < 0.05; ** *p* < 0.01; *** *p* < 0.001

^a^189 out of 200 amino acids analyzed in human–chimp comparison

^b^376 out of 383 amino acids analyzed in human–chimp comparison

^c^196 out of 202 amino acids analyzed in human–chimp comparison

^d^119 out of 186 amino acids analyzed in human–chimp comparison

^e^314 out of 387 amino acids analyzed in human–chimp comparison

N.D., not determined

**Figure 4:**
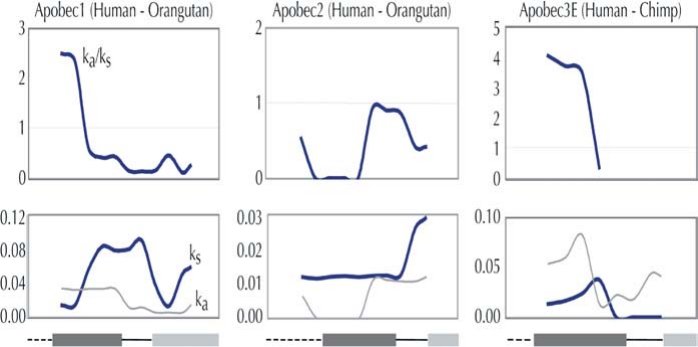
Selective Pressures on APOBEC1, APOBEC2, and APOBEC3E Sliding window analysis (250-bp window; 50-bp slide) was performed on three *APOBEC* genes. Although *APOBEC1* demonstrates purifying selection when the whole gene is analyzed (Table 1), the sliding window analysis of a human–orangutan comparison reveals a window (aa 1–100) in the first active site (dark gray bar), which shows evidence of positive selection (*p* < 0.01). Sliding window analysis of APOBEC2, which is also evolving under purifying selection (Table 1), does not show any windows where ω is greater than one. APOBEC3E, which gives the strongest signal for positive selection (Table 1), has ω greater than one for almost all windows. (Note that ω is not plotted where Ks = 0). DOI:10.1371/journal.pbio.0020275.g004

ω ratios were calculated for human–chimp orthologs, and tested against the neutral expectation that ω = 1 (*p*-values obtained from simulations performed in K-estimator). Values of ω significantly less and greater than one imply purifying and positive/diversifying selection, respectively. We were unable to obtain enough *APOBEC2* sequence from the chimpanzee genome project to do this analysis, so *APOBEC2* was sequenced from orangutan. When sliding window analysis was performed, *APOBEC1* (human–orangutan; see Figure 4), *APOBEC3C* (human–gorilla), and *APOBEC3F* (human–chimp) show regions of both significant positive and purifying selection. Windows of positive selection in these genes are indicated as amino acid ranges (e.g., aa 1–100 for APOBEC1) along with the associated ω values and statistical significance

DOI:10.1371/journal.pbio.0020275.t001

The limited divergence between human and chimp genomes leads to some comparisons not being informative enough to detect selection *(APOBEC1* and *APOBEC3C),* and there was insufficient chimpanzee sequence available in one case *(APOBEC2).* To gain further information about these genes, we sequenced them from either orangutan or gorilla (Table 1, third column). These comparisons reveal that strong purifying selection has acted on *APOBEC2,* but positive selection can be detected in both *APOBEC1* (aa 1–100; also see Figure 4) and *APOBEC3C* (aa 34–133). Although we might have expected *APOBEC1* to be evolving only under purifying selection based on its important editing of *APOB* mRNA, our analysis suggests that *APOBEC1* has also participated in some kind of genetic conflict involving its first active site, and suggests that the rapid evolution of *APOBEC1* seen previously in mouse–rat comparisons may also be due to positive selection (Nakamuta et al. 1995). Figure 4 shows representative sliding window analyses of genes undergoing gene-wide purifying *(APOBEC2)* and positive *(APOBEC3E)* selection. These findings greatly extend the current understanding of the *APOBEC* family, and implicate a majority of *APOBEC* genes as participants in host defense. They also raise the possibility of other editing systems being involved in genome defense; for instance, hepatitis delta virus is known to be edited by adenosine deaminase (Polson et al. 1996).

### Human APOBEC3G Polymorphisms and AIDS

The antiviral activity of APOBEC3G and the excess of non-synonymous changes specific to human *APOBEC3G* (see Figure 2) implicate non-synonymous polymorphisms as being functionally very important. Because binding by Vif inhibits APOBEC3G's antiviral ability, we might predict that *APOBEC3G* should be subject to overdominant selection (heterozygous individuals being at a selective advantage), especially in populations with a high incidence of HIV infection, since different alleles of *APOBEC3G* may have different susceptibility to various viral strains. The action of APOBEC3G on viral evolution could also be complex because, although it is ineffective as an antiviral mechanism in the presence of Vif, its action could also result in an increased likelihood of adaptive changes and viral diversity in the host due to the introduced G-to-A hypermutations. Polymorphisms in *APOBEC3G* may thus have direct impact on the progression time from initial HIV infection to AIDS, and should be investigated as such.

### What Drives the Long-Term Evolution of APOBEC3G?

The evidence for positive selection of *APOBEC3G* does not identify the biological step that exerts this selective pressure. Formally, this step could be the yet-undefined mechanism by which APOBEC3G is packaged into virions, the interaction of APOBEC3G with Vif-like destruction proteins encoded by other viruses, and/or its interaction with the proteasome machinery. APOBEC3G may indeed interact with other viruses, because G-to-A hypermutation—a hallmark of the single-stranded DNA–editing activity of APOBEC3G-like enzymes—has been observed in some nonlentivirus viruses (Vartanian et al. 2003), and because APOBEC3G has recently been shown to inhibit the replication of the hepatitis B virus upon deliberate coexpression (Turelli et al. 2004). However, this inhibition of hepatitis B is not correlated with G-to-A hypermutation, suggesting that APOBEC3G may also inhibit viral replication independent of its catalytic activity.

The ancient, constant pressure of positive selection on *APOBEC3G* in primates raises the possibility that at least some of its evolution may be explained by a struggle not in the lymphocytes, but in the germline, where *APOBEC3G* is also abundantly expressed (Jarmuz et al. 2002), and where genome-restricted mobile genetic elements need to transpose to ensure survival. Of the three main classes of eukaryotic mobile elements, only two are active in humans and, most likely, other primate genomes. The first and major class includes the LINE1 (long interspersed element–1) non-LTR (long terminal repeat) retroposons that are not a likely target for APOBEC3G, because they carry out their reverse transcription in the nucleus (APOBEC3G is restricted to the cytoplasm). A second class, the LTR-bearing human endogenous retroviruses (HERVs), is identical in many aspects of its life cycle to retroviruses. While the selective disadvantage to an individual organism conferred by endogenous retroviruses may pale in comparison to that of pathogenic viruses, over time the steady retrotransposition of endogenous retroviruses is likely to be more detrimental to a species than scattered, episodic interactions with viruses. Thus, the constant efforts of HERVs to jockey for evolutionary dominance may provide a more likely explanation for the positive selection of *APOBEC3G* and other *APOBEC* genes in primate genomes.

## Materials and Methods

### 

#### Genomic DNA sequencing of primate samples.

Genomic DNA was obtained from Coriell (Camden, New Jersey, United States). Species and Coriell repository numbers are: Pan troglodytes (chimpanzee) (NAO3448A), Pan paniscus (bonobo) (NGO5253), Gorilla gorilla (gorilla) (NG05251B), Pongo pygmaeus (orangutan) (NAO4272), Macaca nigra (Celebes crested macaque) (NG07101), Macaca fascicularis (crab-eating macaque) (NA03446), Erythrocebus patas (patas monkey) (NG06254), Lagothrix lagotricha (common woolly monkey) (NG05356), and Saguinus labiatus (red-chested mustached tamarin) (NG05308). Papio anubis (baboon) DNA was a personal gift from Dr. Trent Colbert. The *APOBEC3G, APOBEC1, APOBEC2,* and *APOBEC3C* genes were amplified exon-by-exon from genomic DNA with PCR Supermix High Fidelity (Invitrogen, Carlsbad, California, United States), and PCR products were sequenced directly. PCR and sequencing primers are shown in Table S1. The human *APOBEC3G* sequence was obtained from the Ensembl database of the human genome project (ENSG00000100289). The Chlorocebus aethiops (African green monkey) *APOBEC3G* sequence (GenBank AY331714.1) is missing the last 21 bp of the coding sequence because it was sequenced from mRNA (Mariani et al. 2003) in a previous study. Exon–intron boundaries are conserved, except in *APOBEC3G* from NWMs (woolly monkey and tamarin) where the “AG” directly 5′ of the eighth coding exon is missing. Sequences have been deposited in GenBank under the following accession numbers: *APOBEC3G* (AY622514–AY622593), *APOBEC3C* (AY622594–AY622597), *APOBEC2* (AY622598–AY622599), *APOBEC1* (AY622600–AY622604).

#### Sequences of other *APOBEC* family members.

Human sequences were obtained from the Ensembl or GenBank databases: *APOBEC1* (ENSG00000111701), *APOBEC2* (ENSG00000124701), *AID* (ENSG00000111732), *APOBEC3A* (ENSG00000128383), *APOBEC3B* (NM_004900.3), *APOBEC3C* (ENSG00000179750), *APOBEC3DE* (ENSG00000179007), and *APOBEC3F* (ENSG00000128394). Transcripts for both *APOBEC3D* (NM_152426) and *APOBEC3DE* (BC017022.1) exist in the database. Chimp sequences were obtained from orthology to human genes assigned on the University of California at Santa Cruz Genome Bioinformatics Website (http://www.genome.ucsc.edu). All orthologous chimp exons were checked for AG and GT flanking the 5′ and 3′ boundaries, respectively, an indication that human splice sites are conserved. The mouse APOBEC3 protein sequence can be found in GenBank (NP_084531.1).

#### Sequence analysis.

DNA sequences were aligned using Clustal_X (Thompson et al. 1997), with hand alignment of small indels based on amino acid sequence. Changes along each lineage (see Figure 2) were assigned using parsimony and counted by hand. Changes at 18 positions could not be unambiguously assigned as non-synonymous or synonymous and were excluded from the R:S ratios. Ka and Ks for pairwise comparisons (Figures 3A–3C and 4; Table 1), as well as their confidence values, were calculated using the K-estimator software package (Comeron 1999). For confidence values, simulations were carried out under the condition where Ka equals Ks and compared to actual Ka from that region, and multiple parameters for transition:transversion ratios were simulated.

Maximum likelihood analysis was performed with the PAML software package (Yang 1997). Global ω ratios for the tree (see Figure 2) were calculated by a free-ratio model, which allows ω to vary along different branches. To detect selection, the multiple alignments were fitted to either the F3×4 or F61 models of codon frequencies. We then compared the log-likelihood ratios of the data using different NSsites models: model 1 (two-state, neutral, ω > 1 disallowed) to model 2 (similar to model 1 but ω >1 allowed), and model 7 (fit to a beta distribution, ω > 1 disallowed) to model 8 (similar to model 7 but ω >1 allowed). In both cases, permitting sites to evolve under positive selection gave a much better fit to the data (*p* < 10^−13^) with a significant fraction of the sites (more than 30%) predicted to evolve at average ω ratios greater than 3.5 (see Figure S2 for details). These analyses also identified certain amino acid residues with high posterior probabilities (greater than 0.95) of having evolved under positive selection (Figures 3D and S2).

## Supporting Information

Figure S1 Episodic Evolution of APOBEC3G Protein Domains The evolutionary history of APOBEC3G is represented. R:S ratios are indicated along each branch of the primate cladogram. The N-terminal domain (A) has undergone adaptive evolution in at least three distinct periods. Despite being only 29 codons long, this domain has accumulated ten non-synonymous changes and only two synonymous changes in the African green monkey since it and the patas monkey last shared a common ancestor. Similarly, the orangutan has retained eight non-synonymous changes and no synonymous changes since it split from the rest of the hominids. Finally, a ratio of 6:0 R:S changes is seen in the split between the NWMs and the common ancestor of OWMs and hominids. Surprisingly, even the two active site structures of APOBEC3G (B and E) show evidence for adaptive evolution (despite all the putative catalytic residues being conserved), including along the branch leading to the common ancestor of all hominids. The first pseudoactive domain (D) acquired ten non-synonymous and no synonymous changes since the hominids split from the OWMs.
(343 KB PDF).

Figure S2 PAML Analysis of APOBEC3G Maximum likelihood analysis was performed on APOBEC3G sequences using the PAML software package. To detect selection, the multiple alignments were fitted to either the F3×4 (A) or F61 (B) models of codon frequencies. We compared the log-likelihood ratios of the data using comparisons of different NSsites models: model 1 (two-state, neutral, ω > 1 disallowed) versus model 2 (similar to model 1 but ω > 1 allowed) and model 7 (fit to a beta distribution, ω > 1 disallowed) versus model 8 (similar to model 7 but ω > 1 allowed). In both cases, permitting sites to evolve under positive selection gave a much better fit to the data (*p* <10^−13^) (C) with a significant fraction of the sites (more than 30%) predicted to evolve at average ω ratios greater than 3.5. These analyses also identified certain amino acid residues with high posterior probabilities (greater than 0.95) of having evolved under positive selection (A and B).
(60KB PDF).

Figure S3 Alignment of APOBEC3G Protein Sequences The individual domains of the APOBEC3G protein are demarcated. Catalytically important residues are highlighted in bold, and those residues identified by PAML analysis as being under positive selection are indicated with gray shading. Blue shading highlights the single amino acid residue that can switch specificity of Vif interaction with APOBEC3G. AGM, African green monkey.
(46 KB PDF).

Table S1 Complete List of Primers Used in This Study (39 KB PDF).

### Accession numbers

The GenBank (http://www.ncbi.nlm.nih.gov/) and Ensembl (http://www.ensembl.org/) accession numbers for the genes and gene products discussed in this paper are as follows. GenBank: *APOBEC1* (AY622600–AY622604), *APOBEC2* (AY622598–AY622599), mouse *APOBEC3* (NP_084531.1), human *APOBEC3B* (NM_004900.3), *APOBEC3C* (AY622594–AY622597), *APOBEC3D* (NM_152426), *APOBEC3DE* (BC017022.1), *APOBEC3G* (AY622514–AY622593), and African green monkey *APOBEC3G* (AY331714.1). Ensembl (all human sequences): *APOBEC1* (ENSG00000111701), *APOBEC2* (ENSG00000124701), *AID* (ENSG00000111732), *APOBEC3A* (ENSG00000128383), *APOBEC3C* (ENSG00000179750), *APOBEC3DE* (ENSG00000179007), *APOBEC3F* (ENSG00000128394), and *APOBEC3G* (ENSG00000100289).

Coriell (http://www.coriell.undmj.edu/) repository numbers for primate genomic DNAs are Pan troglodytes (chimpanzee) (NAO3448A), Pan paniscus (bonobo) (NGO5253), Gorilla gorilla (gorilla) (NG05251B), Pongo pygmaeus (orangutan) (NAO4272), Macaca nigra (Celebes crested macaque) (NG07101), Macaca fascicularis (long-tailed macaque) (NA03446), Erythrocebus patas (patas monkey) (NG06254), Lagothrix lagotricha (common woolly monkey) (NG05356), and Saguinus labiatus (red-chested mustached tamarin) (NG05308).

## Abbreviations

aa: amino acids
AID: activation-induced deaminase
APOB: apolipoprotein B
APOBEC: apolipoprotein B–editing catalytic polypeptide
G-to-A: guanine-to-adenine
Ka: number of substitutions per non-synonymous site
Ks: number of substitutions per synonymous site
LTR: long terminal repeat
NWM: New World monkey
OWM: Old World monkey
PAML: phylogenetic analysis by maximum likelihood
R:S: ratio of non-synonymous to synonymous changes
SIV: simian immunodeficiency virus
Vif: virion infectivity factor
ω: Ka/Ks ratio
