# Supplementary material for: Ancient Adaptive Evolution of the Primate Antiviral DNA-Editing Enzyme APOBEC3G
Source: PLoS Biol. 2004 Jul 20;2(9):e275. doi: 10.1371/journal.pbio.0020275 (PMC479043; doi:10.1371/journal.pbio.0020275)
Supplement: Figure S1 — The evolutionary history of APOBEC3G is represented. R:S ratios are indicated along each branch of the primate cladogram. The N-terminal domain (A) has undergone adaptive evolution in at least three distinct periods. Despite being only 29 codons long, this domain has accumulated ten non-synonymous changes and only two synonymous changes in the African green monkey since it and the patas monkey last shared a common ancestor. Similarly, the orangutan has retained eight non-synonymous changes and no synonymous changes since it split from the rest of the hominids. Finally, a ratio of 6:0 R:S changes is seen in the split between the NWMs and the common ancestor of OWMs and hominids. Surprisingly, even the two active site structures of APOBEC3G (B and E) show evidence for adaptive evolution (despite all the putative catalytic residues being conserved), including along the branch leading to the common ancestor of all hominids. The first pseudoactive domain (D) acquired ten non-synonymous and no synonymous changes since the hominids split from the OWMs. (343 KB PDF). [file pbio.0020275.sg001.pdf]

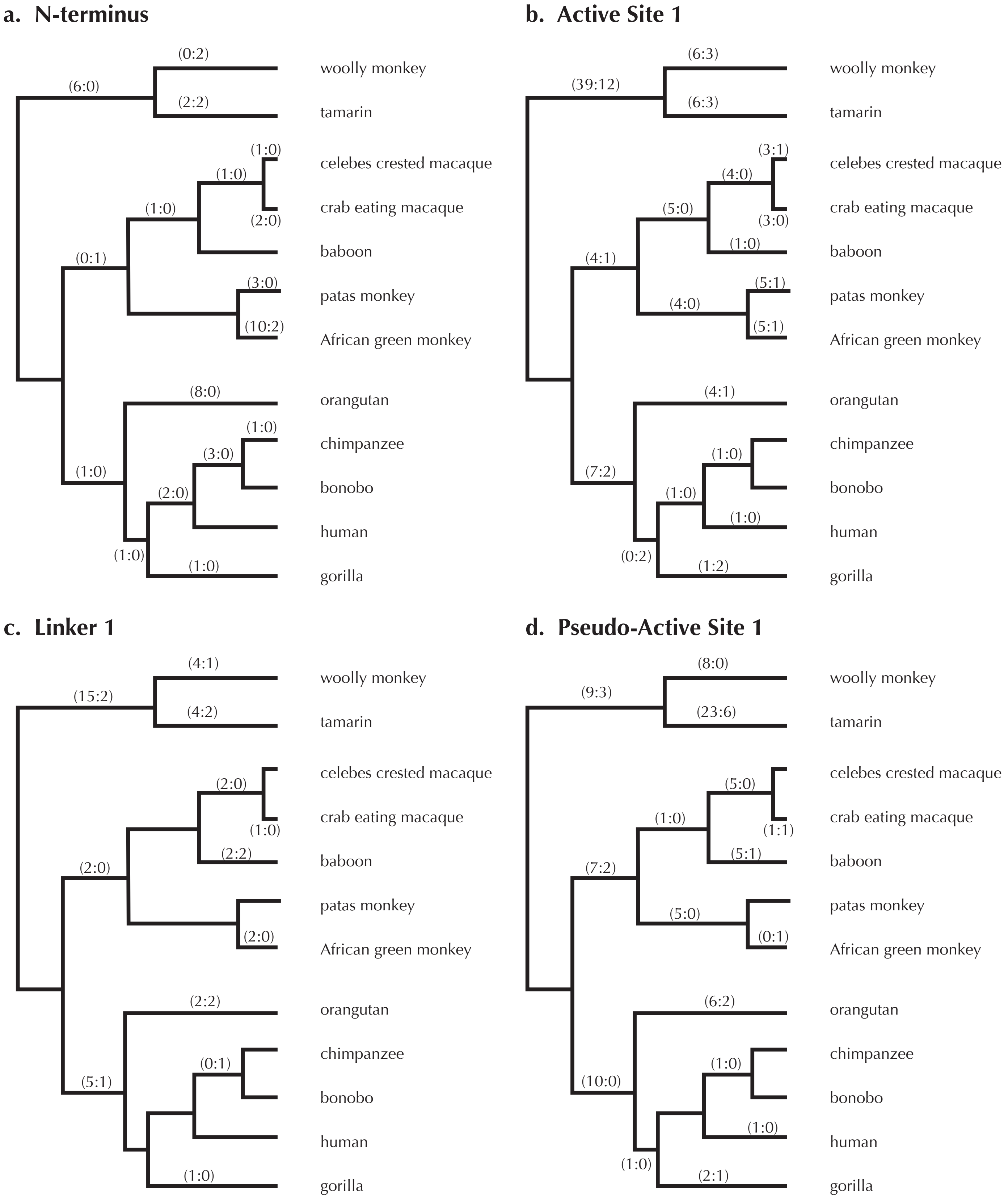

Figure S2
Sawyer et al.

e. Active Site 2

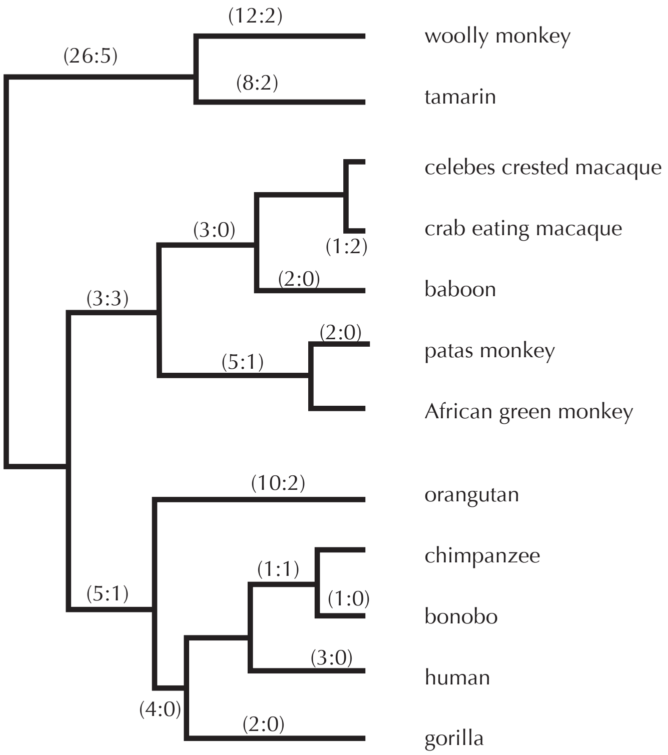

f. Linker 2

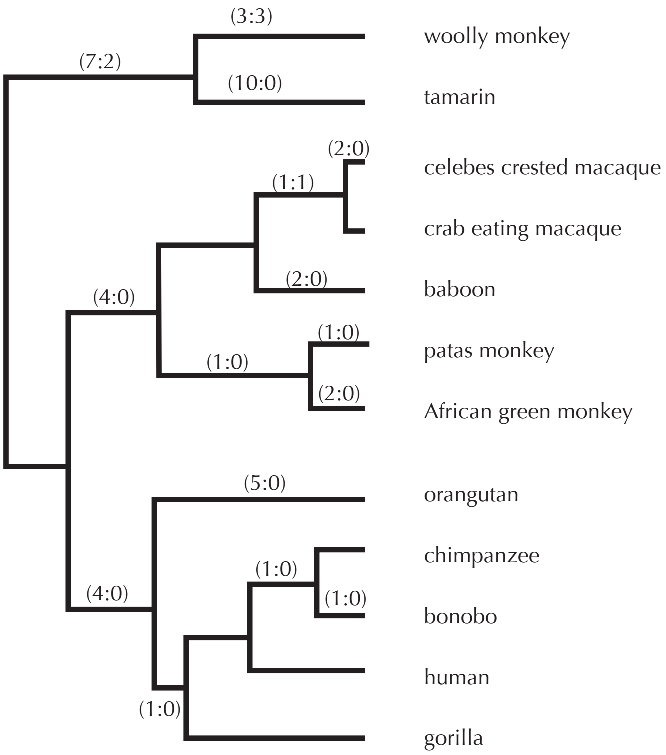

g. Pseudo-Active Site 2

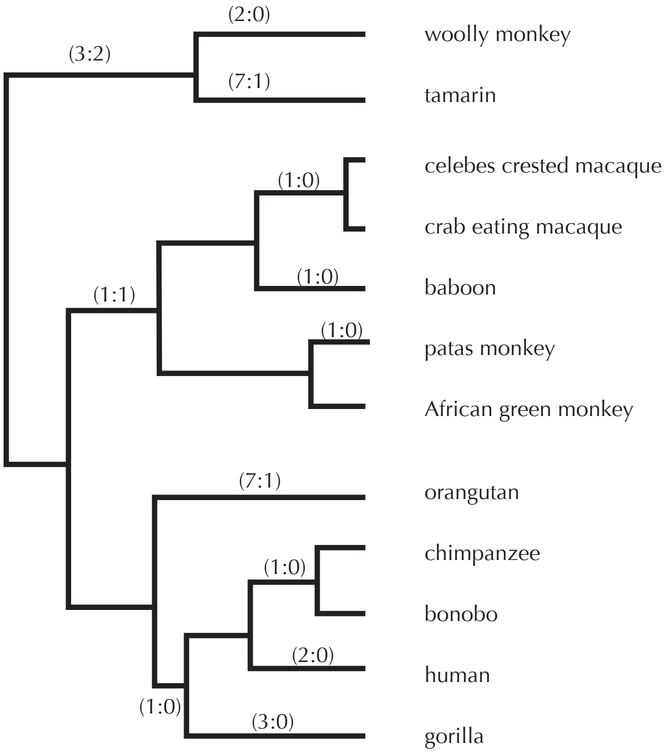

Figure S2 (cont.)
Sawyer et al.
