# Supplementary material for: Ancient Adaptive Evolution of the Primate Antiviral DNA-Editing Enzyme APOBEC3G
Source: PLoS Biol. 2004 Jul 20;2(9):e275. doi: 10.1371/journal.pbio.0020275 (PMC479043; doi:10.1371/journal.pbio.0020275)
Supplement: Figure S2 — Maximum likelihood analysis was performed on APOBEC3G sequences using the PAML software package. To detect selection, the multiple alignments were fitted to either the F3×4 (A) or F61 (B) models of codon frequencies. We compared the log-likelihood ratios of the data using comparisons of different NSsites models: model 1 (two-state, neutral, ω > 1 disallowed) versus model 2 (similar to model 1 but ω > 1 allowed) and model 7 (fit to a beta distribution, ω > 1 disallowed) versus model 8 (similar to model 7 but ω > 1 allowed). In both cases, permitting sites to evolve under positive selection gave a much better fit to the data (p <10−13) (C) with a significant fraction of the sites (more than 30%) predicted to evolve at average ω ratios greater than 3.5. These analyses also identified certain amino acid residues with high posterior probabilities (greater than 0.95) of having evolved under positive selection (A and B). (60KB PDF). [file pbio.0020275.sg002.pdf]

A. Log Likelihood Scores and Parameter Estimates for Four Models of Variable ω's Among Sites Assuming the F3x4 Model of Codon Frequencies

Model	Parameter Estimates	Sites* with ω > 1	ℓ
M0: one ratio	average dN/dS for each branch = 1.316		**-4209.047**
**Site models**			
M1: neutral	(ω = 0) *f* = 0.3275<br>(ω = 1) (*f* = 0.6275)<br>average dN/dS for each branch = 0.6725	Not allowed	**-4171.503**
M2: selection	(ω = 0) *f* = 0.292<br>(ω = 1) *f* = 0.379<br>**ω = 3.777**(*f* = 0.329)<br>average dN/dS for each branch = 1.6203	8, 13, 14, 29, **59,** 61, 69, 102, 169, 187, 196, 200, **213**, 248, **249**, **274, 276**, 303, 330, **344**, 365	**-4136.417**
M3:discrete	ω = 0.00001 *f* = 0.31982<br>**ω = 1.305 (*f* = 0.4178)**<br>**ω = 4.189 (*f* = 0.2624)**<br>average dN/dS for each branch = 1.6444	Too many to list	**-4136.302**
M7:Beta	*p* = 0.00397 *q* = 0.00186<br>average dN/dS for each branch = 0.7000	Not allowed	**-4171.826**
M8:Beta&ω	*p* = 0.02755 *q* = 0.02328<br>*f* = 0.658<br>**ω = 3.702 (*f* = 0.342)**<br>average dN/dS for each branch = 1.6169	8, 13, 14, 29, **59,** 61, 69, 99, 102, 169, 176, 187, 196, 200, **213**, 248, **249**, **274, 276**, 303, 330, **344**, 362, 365	**-4136.495**

B. Log Likelihood Scores and Parameter Estimates for Four Models of Variable ω's Among Sites Assuming the F61 Model of Codon Frequencies

Model	Parameter Estimates	Sites* with ω > 1	ℓ
M0: one ratio	average dN/dS for each branch = 1.3078		**-4152.141**
**Site models**			
M1: neutral	(ω = 0) *f* = 0.31758<br>(ω = 1) (*f* = 0.68242)<br>average dN/dS for each branch = 0.6824	Not allowed	**-4116.327**
M2: selection	(ω = 0) *f* = 0.27762<br>(ω = 1) *f* = 0.40166<br>**ω = 3.745**(*f* = 0.3207)<br>average dN/dS for each branch = 1.6029	5, 8, 13, 14, 29, 59**,** 61, 102, 169, 187, 196, 200, **213**, 248, 249, **274, 276**, 303, 330, **344**	**-4084.584**
M3:discrete	ω = 0.00001 *f* = 0.3048<br>**ω = 1.292 (*f* = 0.44)**<br>**ω = 4.184 (*f* = 0.255)**<br>average dN/dS for each branch = 1.6366	Too many to list	**-4084.419**
M7:Beta	*p* = 0.00289 *q* = 0.00106<br>average dN/dS for each branch = 0.7	Not allowed	**-4116.456**
M8:Beta&ω	*p* = 0.00223 *q* = 0.00136<br>*f* = 0.6835<br>**ω = 4.43 (*f* = 0.3165)**<br>average dN/dS for each branch = 1.606	5, 8, 13, 14, 29, 59**,** 61, 102, 169, 187, 196, 200, **213**, 248, 249, **274, 276**, 303, 330, **344**	**-4084.590**

Note: *p* and *q* are parameters of the beta distribution. *f* is the proportion of sites assigned to an individual ω category or to a beta distribution with shape parameters *p* and *q*. The proportion *f* in parentheses is not a free parameter. Sites assigned to ω are those with posterior probabilities (*P*) > 0.95 , and those with *P* > 0.99 are in bold. Note: analyses conducted using ω as a free parameter.

*Codon positions listed have been adjusted to correspond to the human Apobec3G sequence, not to the multiple alignment where small gapped regions had to be removed prior to performing this analysis. Note that the species-specific residue (128) does not show up as being under positive selection. This is because it has not repeatedly changed.

C. Likelihood ratio test statistics (2[illegible]) for models of variable selective pressure among sites.

	2[illegible]	df	*P*-value
**F3x4**			
One ratio vs. M3 (*k* = 3)	145.49	4	***P* < 0.0001**
M1 vs. M2	70.172	2	***P* < 0.0001**
M7 vs. M8	70.662	2	***P* < 0.0001**
**F61**			
One ratio vs. M3 (*k* = 3)	135.444	4	***P* < 0.0001**
M1 vs. M2	63.486	2	***P* < 0.0001**
M7 vs. M8	63.732	2	***P* < 0.0001**

Figure S1
Sawyer et al.
