# Supplementary material for: Ancient Adaptive Evolution of the Primate Antiviral DNA-Editing Enzyme APOBEC3G
Source: PLoS Biol. 2004 Jul 20;2(9):e275. doi: 10.1371/journal.pbio.0020275 (PMC479043; doi:10.1371/journal.pbio.0020275)
Supplement: Figure S3 — The individual domains of the APOBEC3G protein are demarcated. Catalytically important residues are highlighted in bold, and those residues identified by PAML analysis as being under positive selection are indicated with gray shading. Blue shading highlights the single amino acid residue that can switch specificity of Vif interaction with APOBEC3G. AGM, African green monkey. (46 KB PDF). [file pbio.0020275.sg003.pdf]

Figure S3
Sawyer et al.

```
Human aa:       1  (N-terminus)               (Active Site 1)               58
AGM             MNPQIRNMVEQMEPDIFVYYFNNRPILSG|RNTVWLCYEVK--TKDPSGPPLDANIFQGKL
Patas           MKPHFRNTVERMYRGTFFYNFNNRPILSR|RNTVWLCYEVK--TRGPSMPTWGAKIFRGQL
CrestMac        MKPQFRNTVERMYRGTFFYSFNNRPILSR|RNTVWLCYEVK--TRGPSMPTWGTKIFRGQV
CrabEatMac      MQPQYRNTVERMYRGTFFYNFNNRPILSR|RNTVWLCYEVK--TRGPSMPTWDTKIFRGQV
Baboon          MKPQFRNTVERMYRDTFFYNFNNRPILSR|RNTVWLCYEVK--TRGPSMPTWDAKIFRGQV
Chimp           MKPHFRNPVERMYQDTFSDNFYNRPILSH|RNTVWLCYEVK--TKGPSRPPLDAKIFRGQV
Bonobo          MKPHFRNPVERMYQDTFSDNFYNRPILSR|RNTVWLCYEVK--TKGPSRPPLDAKIFRGQV
Human           MKPHFRNTVERMYRDTFSYNFYNRPILSR|RNTVWLCYEVK--TKGPSRPPLDAKIFRGQV
Gorilla         MTPQFRNTVERMYRDTFSYNFNNRPILSR|RNTVWLCYEVK--TKDPSRPPLDAKIFRGQV
Orangutan       MNPQFRNMVDGMDPHKFSYNFKNRPILSR|RNTVWLCYEVK--TKGPSRPPLDAKIFRGQV
WoollyMon       MKPQTRNTVVRMDPDTFFYNFYNRPILSH|RNTVWLCYEVKMKTNDPSRPPLVANIFQGQV
Tamarin         MKPQTRNTVVRMDPDTFFYDFYNRPILSD|RNTVWLCYEVKMKTNDRSRPPLVAKILEGQV

Human aa:       59                                                          117
AGM             Y-PEAKDHPEMKFLHWFRKWRQLHRDQEYEVTWYVSWSPCTRCANSVATFLAEDPKVTLT
Patas           Y-PEAKDHPEMKFLHWFRKWRQLHRDQEYEVTWYVSWSPCTRCANSVATFLAEDPKVTLT
CrestMac        Y-SKAKYHPEMRFLRWFSKWRQLHHDQEYKVTWYVSWSPCTRCANSVATFLAKDPKVTLT
CrabEatMac      LRSKAKYHPEMRFLHWFREWRQLHHDQEYKVTWYVSWSPCTRCANSVATFLAKDPKVTLT
Baboon          Y-SKAKYHPEMRFLHWFRKWRQLHRDQEYEVTWYVSWSPCTGCANSVATFLAEDPKVTLT
Chimp           Y-SKLKYHPEMRFFHWFSKWRKLHRDQEYEVTWYISWSPCTKCTRDVATFLAEDPKVTLT
Bonobo          Y-SKLKYHPEMRFFHWFSKWRKLHRDQEYEVTWYISWSPCTKCTRDVATFLAEDPKVTLT
Human           Y-SELKYHPEMRFFHWFSKWRKLHRDQEYEVTWYISWSPCTKCTRDMATFLAEDPKVTLT
Gorilla         Y-SELKYHPEMRFFHWFSKWRKLHRDQEYEVTWYISWSPCTKCTRNVATFLAEDPKVTLT
Orangutan       Y-FELKNHPEMRFFHWFSKWRKLHRDQECEVTWYMSWSPCTKCTRNVATFLAEDPKVTLT
WoollyMon       S-FNPEHHAEMYFLSWFR-GNLLPACKRSQITWFVSWNPCLYCVAKVAEFLAEHPKVTLT
Tamarin         H-FDPEHHAEMYFLSWFR-GNLLQACKSSQITWFVSWNPCLNCVAKVAEFLAEHPNVTLT

                                                          (PseudoActive
Human aa:       118        128     (Linker 1)                Site 1)      177
AGM             IFV|ARLYYFWKPDYQQALRILCQERGGPHATMKIMNYNEFQHCW|NEFVDGQGKPFKPRKN
Patas           IFV|ARLYYFWKPDYQEALRILCQKRGGPHATMKIMNYNEFQHCW|NEFVDGQGKPFKPRKN
CrestMac        IFV|ARLYYFWKPDYQQALRILCQKRGGPHATMKIMNYNEFQDCW|NKFVDGRGKPFKPRNN
CrabEatMac      IFV|ARLYYFWKPDYQQALRILCQKRGGLHATMKIMNYNEFQDCW|NKFVDGGGKPFKPRNN
Baboon          IFV|ARLYYFWKPDYQEALRVLCQKRGSPHATMKIMNYNEFQHCW|NKFVRGRREPFEPWEN
Chimp           IFV|ARLYYFWDPDYQEALRSLCQKRDGPRATMKIMNYDEFQHCW|SKFVYSQRELFEPWNN
Bonobo          IFV|ARLYYFWDPDYQEALRSLCQKRDGPRATMKIMNYDEFQHCW|SKFVYSQRELFEPWNN
Human           IFV|ARLYYFWDPDYQEALRSLCQKRDGPRATMKIMNYDEFQHCW|SKFVYSQRELFEPWNN
Gorilla         IFV|ARLYYFWDQDYQEALRSLCQKRDGPRATMKIMNYDEFQHCW|SKFVYSQRELFEPWNN
Orangutan       IFV|ARLYYFWDPDYQEALRSLCQERDGPRANMKIMNYDEFQHCW|NKFVYSQRELFEPWNN
WoollyMon       VST|ARLYCYRKKDWRRALRKLSQTG----ARVKIMDYEEFQHCW|DNFVDNQREPFEPWNA
Tamarin         VST|ARIYCYWKKDWRRALRKLCQTG----ARVKIMNYKEFAYCW|ENFVYKERKPFRYWDK
```

```
Human aa:       178                                     (Active Site 2)       237
AGM             LPKHYTLLHATLGELLRHVMDPGTFTSNFNNKPWVSG|QRETYLCYKVERSHNDTWVLLNQ
Patas           LPKHYTLLHATLGELLRHVMDPGTFTSNFNNKPWVSG|QRETYLCYKVERSHNDTWVLLNQ
CrestMac        LPKHYTLLQATLGELLRHLMDPGTFTSNFNNKPWVSG|QHETYLCYKVERLHNDTWVPLNQ
CrabEatMac      LPKHYTLLQATLGELLRHLMDPGTFTSNFNNKPWVSG|QHETYLCYKVERLHNDTWVPLNQ
Baboon          LPKHYTLLHATLGELLRHLMDPGTFTSNFYNKPWVSG|QHETYLCYKVERLHNGTWVPLNQ
Chimp           LPKYYILLHIMLGEILRHSMDPPTFTSNFNNELWVRG|RHETYLCYEVERLHNDTWVLLNQ
Bonobo          LPKYYILLHIMLGEILRHSMDPPTFTSNFNNELWVRG|RHETYLCYEVERLHNDTRVLLNQ
Human           LPKYYILLHIMLGEILRHSMDPPTFTFNFNNEPWVRG|RHETYLCYEVERMHNDTWVLLNQ
Gorilla         LPKYYMLLHIMLGEILRHSMDPPTFTSNFNNEHWVRG|RHETYLCYEVERLHNDTWVLLNQ
Orangutan       LPKYYIVLHIILGEILRHSMDPLTFTSNFNNEPCVEG|RHETYLCYKVERLHNDTWVLLNQ
WoollyMon       LPKHYTLLRITLGEVLRHRMDPVTFTYNFTNDPSVLG|QHQSYLCYKVEHLRNGTWVPLHQ
Tamarin         FSGNYRFLRCKLQEILRHLMDPGTFTYNFTNDPSVLG|RHQTYLCYEAEHLHSGTWVPLHQ

Human aa:       238                                                          297
AGM             HRGFLRNQAPDRHGFPKGRHAELCFLDLIPFWKLD-DQQYRVTCFTSWSPCFSCAQKMAK
Patas           HRGFLRNQAPDRHGFPKGRHAELCFLDLIPFWKLD-DQQYRVTCFTSWSPCFSCAQKMAK
CrestMac        HRGFLRNQAPNIHGFPKGRHAELCFLDLIPFWKLD-GQQYRVTCFTSWSPCFSCAQEMAK
CrabEatMac      HRGFLRNQAPNIHGFPKGRHAELCFLDLIPFWKLD-GQQYRVTCFTSWSPCFSCAQEMAK
Baboon          HRGFLRNQAPDIHGFPKGRHAELCFLDLIPFWKLD-GQQYRVTCFTSWSPCFSCAQEMAK
Chimp           RRGFLCNQAPHKHGFLEGRHAELCFLDVIPFWKLDLHQDYRVTCFTSWSPCFSCAQEMAK
Bonobo          RRGFLCNQAPHKHGFLEGRHAELCFLDVIPFWKLDLHQDYRVTCFTSWSPCFSCAQEMAK
Human           RRGFLCNQAPHKHGFLEGRHAELCFLDVIPFWKLDLDQDYRVTCFTSWSPCFSCAQEMAK
Gorilla         RRGFLCNQAPHKHGFLEGRHAELCFLDVIPFWKLDLHQDYRVTCFTSWSPCFSCAQEMAK
Orangutan       RRGFLCNQAPAIHGFPEGRHAELCFLDVIPFWKLDGKQRYRVTCFTSWSPCFRCAQEMAK
WoollyMon       HRGFILNEASNSVSFPEGRHAELCLLDLISFWKLKQAQRYRVTCFISWSPCFSCAEKVAE
Tamarin         HRGFILNEASNNLSFPEGRHAELCLLDLISFWKLDPAQTYRVTCFISWSPCFSCAQEVAE

                                                                 (Pseudo-Active
Human aa:       298             (Linker 2)                        Site 2) 357
AGM             FISNNKHVSLCIFA|ARIYDDQGRCQEGLRTLHRDGAKIAVMNYSEFEYCW|DTFVDRQGRP
Patas           FISKKKHVSLCIFA|ARIYDDQGRRQEGLRTLHRDGAKIAVMNYSEFKHCW|DTFVDHQGRP
CrestMac        FISNNEHVSLCIFA|ARIYDDQGRYQEGLRTLHRDGAKIAMMNYSEFEYCW|DTFVDRQGRP
CrabEatMac      FISNNEHLSLCIFA|ARIYDDQGRYQEGLRTLHRDGAKIAMMNYSEFKHCW|DTFVDRQGRP
Baboon          FISNNEHVSLCIFA|ARIYDDQGRCQEGLRTLHRDGAKIAMMNYSEFEYCW|DTFVDRQGRP
Chimp           FISNNKHVSLCIFA|ARIYDDQGRCQEGLRTLAKAGAKISIMTYSEFKHCW|DTFVDHQGCP
Bonobo          FISNNKHVSLCIFA|ARIYDDQGRCQEGLRTLAKAGAEISIMTYSEFKHCW|DTFVDHQGCP
Human           FISKNKHVSLCIFT|ARIYDDQGRCQEGLRTLAEAGAKISIMTYSEFKHCW|DTFVDHQGCP
Gorilla         FISNKKHVSLCIFA|ARIYDDQGRCQEGLRTLAEAGAKISIMTYSEFKHCW|DTFVYHQGCP
Orangutan       FISNNQHVSLCIFA|ARIYDDQGRCKEGLRTLDEAEAKISIMTYDEFQHCW|DTFVDHQGRP
WoollyMon       FLQENPHVNLHISA|ARIYDYQRGYKKGLRRLDRAGTPISMMKYSEFKHCW|DTFVDHQGHP
Tamarin         FLHENPHVNLRIFA|ARIYDYRPGYEEGLLRLSWAGAPISMMKYSGFSHCW|DTFVDHQGRS
```

```
Human aa:       358                       385
AGM             FQPWDGLDEHSQALSGRLRAI------
Patas           FQPWDGLDEHSQALSGRLRAILQNQGN
CrestMac        FQPWDGLDEHSQALSERLRAILQNQGN
CrabEatMac      FQPWDGLDEHSQALSERLRAILQNQGN
Baboon          FQPWDGLDEHSQDLSGRLRAILQNQGN
Chimp           FQPWDGLEEHSQALSGRLRAILQNQGN
Bonobo          FQPWDGLEEHSQALSGRLRAILQNQGN
Human           FQPWDGLDEHSQDLSGRLRAILQNQEN
Gorilla         FQPWDGLEEHSQALSGRLQAILQNQGN
Orangutan       FLPWIRLHEHSEALSGRLRAILLNQGN
WoollyMon       FQPWEELNEHSQALSGRLQAILQNQGN
Tamarin         FKPWKGLNEHSQALSGRLQAILQIMGN
```
