# Supplementary material for: Ancient Adaptive Evolution of the Primate Antiviral DNA-Editing Enzyme APOBEC3G
Source: PLoS Biol. 2004 Jul 20;2(9):e275. doi: 10.1371/journal.pbio.0020275 (PMC479043; doi:10.1371/journal.pbio.0020275)
Supplement: Table S1 — (39 KB PDF). [file pbio.0020275.st001.pdf]

Table S1: PCR and Sequencing Strategy (Sawyer et al.)

( ) = PCR primers

* = sequencing primer

underlined = exonic primer, all others in introns

APOBEC3G

	Coding Exon 1	Coding Exon 2	Coding Exon 3	Coding Exon 4	Coding Exon 5	Coding Exon 6	Coding Exon 7	Coding Exon 8
Chimpanzee 3448	(019,020) 071*	(021,022*)	(023,024*)	(004*,007)	(004,007*)	(054,055*)	(003*,006)	(029*,030)
	(019,020) 072*	(040*,042)	(004*,007)		(025,026*)	(054*,055)	(029,030*)	(029,030*)
		(040,042*)					(029*,030)	
Bonobo 5352	(019*,020)	(021,022*)	(004*,007)	(004*,007)	(004,007*)	(054*,055)	(029*,030)	(029,030*)
		(040*,042)	(023*,024)		(025,026*)	(054,055*)	(047*,048)	
		(040,042*)	(023,024*)				(047,048*)	
Gorilla 5251	(019,020*)	(021,022*)	(023,024*)	(004*,007)	(004,007*)	(054,055*)	(047,048*)	(029*,030)
	(019*,020)	(040*,042)	(004*,007)		(025,026*)	(054*,055)	(047*,048)	
		(040,042*)					(029*,030)	
Orangutan 4272	(019*,020)	(040,042*)	(023*,024)	(004*,007)	(025,026*)	(054*,055)	(047,048*)	(047*,049)
		(041,043*)	(023,024*)			(054,055*)	(047,049) 048*	(047,049) 056*
		(040*,042)	(004*,007)				(047*,048)	(029*,030)
		(021,022*)					(047,049*)	(047,050*)
		(041*,043)					(029*,030)	
							(047*,049)	
Crab Eating Macaq 3446	(019*,020)	(040,042*)	(004*,015)	(004*,015)	(004,015*)	(054,055*)	(047,049) 048*	(047,050) 056*
		(040*,042)	(023*,024)		(025,026*)	(054*,055)	(047,048*)	(047,050) 030*
		(041,043*)	(026,024*)		(004,015) 014*		(047*,049)	(047,050*)
		(041*,043)					(047*,048)	
							(047,050) 030*	
							(047,050*)	
Celebes Crested Macaq 7101	(019*,020)	(041,043*)	(023,024*)	(004*,015)	(004,015*)	(054*,055)	(047*,048)	(047,050*)
		(041*,043)	(004*,015)		(025,026*)	(054,055*)	(047*,050)	
					(004,015) 014*			
Patas 6254	(019*,020)	(041,043*)	(023*,024)	(004*,015)	(004,015*)	(054*,055)	(047,049*)	(029*,030)
		(041*,043)	(023,024*)		(025,026*)	(054,055*)		(047,049*)
			(004*,015)		(004,015) 014*			
Baboon	(019*,020)	(041*,043)	(023,024*)	(075*,076)	(025,026*)	(054,055*)	(047*,050)	(047,050*)
		(041,043*)		(075,076*)	(004,015) 014*	(054*,055)	(047,050*)	
		(040*,042)			(004,015*)		(047*,048)	
		(040,042*)					(047,048*)	

Woolly 5356	(019*,020)	(041,043*)<br>(041,043) 042*<br>(041*,022)<br>(041,022) 042*<br>(095,096*)<br>(095,096) 097*<br>(095,096) 098*<br>(041,043) 097*<br>(041,043) 098*	(073,094*)<br>(073*,094)	(075*,076)<br>(075,076*)	(077*,078)<br>(077,078*)	(054,055*)<br>(054*,055)	(047*,050)<br>(047,050) 048*	(047,050*)
Tamarin 5308	(019*,020)	(041,043) 042*<br>(041*,043)	(073*,076)<br>(073,091*)<br>(073,094) 091*<br>(073,094*)<br>(073*,091)<br>(073*,094)	(092,076*)<br>(093,076*)<br>(092*,076)<br>(093*,076)<br>(092,076) 093*	(025,026*)	(054,055*)<br>(054*,055)	(047*,050)<br>(047,050*)<br>(047,050*)	(047,050) 056*<br>(047,050*)<br>(047,050*)
**Apobec2**								
	Coding Exon 1	Coding Exon 2						
Orangutan 4272	(123*,124)<br>(123,124) 119*<br>(123,124*)	(120,121*)<br>(120*,121)						
**Apobec1**								
	Coding Exon 1	Coding Exon 2	Coding Exon 3	Coding Exon 4	Coding Exon 5			
Orangutan 4272	(102*,103)<br>(102,103*)	(104*,106)<br>(104,106*)	(108,111) 109*<br>(108*,111)<br>(108,111*)	(112*,113)<br>(112,113*)	(114*,117)<br>(114,117*)			
**Apobec3C**								
	Coding Exon 1	Coding Exon 2	Coding Exon 3	Coding Exon 4				
Gorilla 5251	(037,038*)<br>(037*,038)	(039*,031)<br>(039,031*)	(032,033) 057*<br>(032,033*)<br>(032,033) 058*	(059*,060)<br>(059,060*)<br>(059,060) 080*				

Oligo Name	Sequence
003	GAGACTGAGGCCCATCCTTCAG
004	TGGAGCCCCTGCACAAAGTG
005	GGGTCCCAGAAGTAGTAGAGGCG
006	GGACGGCATGAGACTTACCTGTG
007	ATGCGCTCCACCTCATAACACAG
008	AATGGATCCACGCACATTCGTCT
009	CACCTTGTGCAGGGGCTCCA
010	GTGGCATGTGGACCGCCTC
011	CGGCCAAGGATGAAGCCTCA
012	CCTGTGTTACAAGGTGGAGCGC
013	GCGCTCCACCTTGTAACACAGG
014	TGATGGATCCAGGCACGTTCAC
015	CTGGTTGCGTAGAAAGCC
016	GAGACTGAGGCCCATCCTTCA
017	GCCCATCCTTCAGTTTTCCTGATTC
018	GGCCCGAAGCCTTCCACTC
019	GAGCGGCCTGTCTTTATCAGAGG
020	GACCTGGGCTGGGACAGATGG
021	GTTTAGTGGACATCAGCCCGGAG
022	TCAGGGCCACGGACTTGG
023	CTCATAGCTGCTTGTAGGTGCCAC
024	CCACAGGTATGTGTGGGAAGGAC
025	CCCTTCTGACAGGTGCTAAGGG
026	GCTACAGGAAGCACAGGTGACAGAC
027	GCTACTTGTACACTCCAGCCTGGGC
028	GATCAAGGCCTTTCCCACAGG
029	CCACCACGATCCCACAGC
030	GGAGATGGTGGTGAACAGCCTG
031	GGGCACATCACTTATTGTTGCG
032	CCCTGCACTCCTCCTGCTCC
033	CATGCTGTCCCTCCTGCACC
037	CACACCAGTGCCTGAGCAGG
038	GCTGAGGCCCAGCAGGAC
039	CCAGGCAGCTGTGTTCAGTGG
040	GAGGCCTAGAGCCGTCCAGG
041	GGAGTGCTCTCTACATTGGCTGG
042	AGTGATTGGAGTCCGGGTGG
043	CTGGCTTAGCTCCTGCCTGC
044	GGTTGGGAGTTTGAGACCAGCC
045	GTGGTGCATGCCTGTAATCCTAGC
046	GGCTGACCCCATCCTACTCC
047	GTCTGTCTTGAGAGTCATGGGC
048	CAGGGAACCCAGAGGAGGC
049	GCTCAACCCAGGTCTCTGCC
050	AGGAGTGCATTGCTTTGCTGG
051	GCCACAGATGAAGCCTCAGACCAG
052	CAGGTAGGTCTGGTGCCGTCC
053	CCTGTGCTGGTGCAGCGGG
054	ACCTCATGGCTTGCTTTCTTTTCTAG
055	CCTACTCCAGGCTTCCATTCTCAC
056	CCAGGCCTCCACCATATGCC
057	CCTCCTCCTCCTTCGCCAG
058	CAGCCCCCCACGTCTCAC
059	GGCCCAGGGAGACCCCTC
060	CTCACTGTTTTCTCCTTGTTTTTTCTCAG
071	CCCTGGGAGGTCACTTTAGGG
072	GCTGGGGCAGGGGAAGG
073	TGACCCTGCTCCTCTCCCAG
074	CCCTGAACCTCCCACTTCTCAC
075	CTTGACTGCGTTCTCTCTTCTTTTTCTTAG
076	GCCTGGAGGGAGACCCTCAC
077	GGCTTTTCTCCCACTGTCCACAG
078	CGGGTGGCTGGTGATCAC
080	CAGATTTTAAATATTGTTGGGAAAACTTTGTG
084	GCTGGGACCTGTTCCACAAATG
085	CTCCCCAATAAAAATTATCAACAAGTTGTACTTAC
086	CTGACCCCTGGTGGTTCACAG
087	CCAGGACCAAACACAGCAGGG
088	CATCAAGCATTGGTTTCATCATGG
089	GCCACCATTTATTCTTGATCTTTTTCAG
090	AGCTTCTCAAAGGCCACCCC
091	CTGACCCCTCCACCTCCTCAC
092	GTGAGGAGGTGGAGGGGTCAG
093	ACTCCTTCCTGCTTCTCCATCTCAG
094	CTGAGATGGAGAAGCAGGAAGGAGT

095	GGCTGGTTTCTCTCTTGTGTCTTCAG
096	CAGATTAATTGGAATCTGGGGGGTAC
097	GATCCAGGCAAATTCTTCCGC
098	GTTTTCATTTTCACTTCATAGCACAGCC
099	CCAGGGGGCTGTGTTTAGTGG
100	GAAGGACACACTGGATAGCCCG
101	CATAACTGCCTGAGATGCGGG
102	TCTGCCCAAGCAGGTGGC
103	TGTTCAAGAATACTTGCCAAGCCC
104	TTCTGAGATGATTTGGAGGTTTGG
105	GAGTGTTGTTTGACATAATCATATCTTGGTC
106	CATTGATTCTTGATCAAATTAAATGGGC
107	GGGATTATAGGCGCATGTGCC
108	GGAAAAGATTTCTAAGATCTCTCTAAATGTGGC
109	AGTGTGGGCTGCTTTTTGTATTTCAG
110	GTTGTAAACAATGAAGTTTCTTTGTTTACTGTTTTTAC
111	CCAATCTTCTGGCCTAAAAACAGC
112	GAATGAGCCAAGATTTGTGTTTCCTTAG
113	CATCAACATTTGTGTCCCTAAAGTGACTTAC
114	TTCCAACTGACATTTTTAAATGTTTGTGG
115	CATCCTAAGGATTAAATATAATCCTTTTTTATTCCAG
116	GAATCAGTACACACACGGAATCATCC
117	ATTCTAAATGGCATTCACTCTTTAGTGGG
118	CTGAGCCACAGCCCCTCC
119	ACCCTGACCCACATCTGAGGC
120	TCCTGTCCTTTTGTCTCCTTGACC
121	GAGGCAAAGCCCAGTTGCC
123	AGCCAGATTTAGCTGCTGACAGC
124	CCTGAATTTCCACAGCCTAACCC
